# Perovskite Single Crystals by Vacuum Evaporation Crystallization

**DOI:** 10.1002/advs.202400150

**Published:** 2024-03-29

**Authors:** Dong Liu, Xianyuan Jiang, Hao Wang, Hao Chen, Ying‐Bo Lu, Siyu Dong, Zhijun Ning, Yong Wang, Zhongchen Wu, Zongcheng Ling

**Affiliations:** ^1^ School of Space Science and Physics Institute of Space Sciences Shandong University Weihai 264209 China; ^2^ School of Physical Science and Technology ShanghaiTech University Shanghai 201210 China; ^3^ State Key Laboratory of High Field Laser Physics and CAS Center for Excellence in Ultra‐Intense Laser Science Shanghai Institute of Optics and Fine Mechanics Chinese Academy of Sciences Shanghai 201800 China; ^4^ Center of Materials Science and Optoelectronics Engineering University of Chinese Academy of Sciences Beijing 100049 China; ^5^ The Edward S. Rogers Department of Electrical and Computer Engineering University of Toronto Toronto Ontario M5S 3G4 Canada

**Keywords:** crystal growth, crystallization, low pressure, perovskite single crystals, vacuum evaporation

## Abstract

Perovskite single crystals have attracted tremendous attention owing to their excellent optoelectronic properties and stability compared to typical multicrystal structures. However, the growth of high‐quality perovskite single crystals (PSCs) generally relies on temperature gradients or the introduction of additives to promote crystal growth. In this study, a vacuum evaporation crystallization technique is developed that allows PSCs to be grown under extremely stable conditions at constant temperature and without requiring additives to promote crystal growth. The new method enables the growth of PSCs of unprecedented quality, that is, MAPbBr_3_ single crystals that exhibit an ultranarrow full width at half maximum of 0.00701°, which surpasses that of all previously reported values. In addition, the MAPbBr_3_ single crystals deliver exceptional optoelectronic performance, including a long carrier lifetime of 1006 ns, an ultralow trap‐state density of 3.67 × 10^9^ cm^−3^, and an ultrahigh carrier mobility of 185.86 cm^2^ V^−1^ s^−1^. This method is applicable to various types of PSCs, including organic–inorganic hybrids, fully inorganic structures, and low‐dimensional structures.

## Graphical abstract

A universal and effective vacuum evaporation crystallization (VEC) method is developed for growing high‐quality perovskite single crystals. The method overcomes the adverse effects of temperature fluctuations and auxiliary substances, and is based on the natural volatilization of solvents at chamber pressure lower than the saturated vapor pressure of the solution.

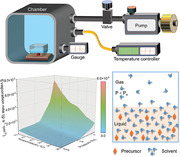

## 1 Introduction

The rapid development of metal halide perovskites (MHPs) has been a significant driving force behind research into single‐crystal applications, including solar cells,^[^ ^1^, ^2^, ^3^, ^4^ ^]^ photodetectors,^[^ ^5^, ^6^, ^7^, ^8^ ^]^ and photomechanical devices.^[^ ^9^, ^10^, ^11^ ^]^ The perovskite single crystals (PSCs) that have been applied in these optoelectronic devices have been grown by various methods, such as solution temperature‐lowering growth (STL),^[^ ^12^ ^]^ inverse temperature crystallization (ITC),^[^ ^13^, ^14^ ^]^ low‐temperature‐gradient crystallization (LTGC),^[^ ^15^ ^]^ antisolvent vapor‐assisted crystallization (AVC),^[^ ^16^ ^]^ liquid‐diffused separation‐induced crystallization (LDSC),^[^ ^17^, ^18^ ^]^ and ligand‐assisted growth (LAG).^[^ ^19^, ^20^ ^]^

Traditionally, supersaturated MHP solutions have been prepared using methods that involve temperature control or the introduction of new substances (such as antisolvents, silicone oil, or ligands).^[^ ^12^, ^13^, ^14^, ^15^, ^16^, ^17^, ^18^, ^19^, ^20^ ^]^ Although these methods have been effective, they introduce certain complexities to the MHP precursors that could potentially affect the quality of the PSCs. For instance, temperature‐controlled crystal growth makes it necessary to maintain a stable temperature gradient and uniform temperature field. This can be challenging because thermal fluctuations and convection in MHP solutions may disrupt the orderly growth of single crystals, which could potentially cause lattice mismatches and dislocations in PSCs.^[^ ^12^, ^13^, ^14^, ^15^, ^21^, ^22^ ^]^ Additionally, the temperature has a limited effect on the solubility of MHPs in solvents.^[^ ^21^ ^]^ Other techniques such as AVC, LDGC, and LAG avoid temperature‐related issues, but require the introduction of additional substances into the MHP precursors.^[^ ^16^, ^17^, ^18^, ^19^, ^20^ ^]^ These substances, although useful in some contexts, can complicate solvent recovery and may influence the quality of PSCs if they become embedded within the cells. An attempt to overcome these problems led to the development of the room‐temperature solvent evaporation‐induced crystallization (RTSEIC) method, a facile approach that relies on the acid‐base reaction between the volatilized solvent and KOH, as well as the hygroscopicity of solid KOH.^[^ ^23^ ^]^ Although the RTSEIC method has been successfully applied to grow various perovskite crystals, it is challenging to accurately control the solvent volatilization process determined by the acid‐base reaction and hygroscopicity of KOH. This is largely because the quantity of KOH progressively decreases throughout the reaction, which complicates precise management of the process. Another attempt entailed the development of vacuum‐flash‐assisted solution processing as a technique to grow perovskite films.^[^ ^24^ ^]^ However, the quality of the perovskite film was not evidently superior to that of the film prepared with the traditional annealing process. One potential problem with this method is that the solvent is rapidly removed under high‐vacuum conditions. Considering these challenges, the exploration of alternative methods for preparing high‐quality PSCs, which avoid temperature gradients and auxiliary substances, may be worthwhile.

Herein, we report a simple and effective method for growing PSCs using vacuum evaporation crystallization (VEC). The high concentration of the supersaturated MHP solution was well maintained by the extremely low‐pressure solvent evaporation technique, and enabled the growth of single crystals under extremely stable conditions. This approach also obviated the need for a temperature gradient and the use of additives. As a result, we successfully produced a MAPbBr_3_ single crystal with an ultranarrow rocking curve of 0.00701°. In comparison, the VEC‐MAPbBr_3_ single crystals exhibit a longer fluorescence lifetime, lower trap density, and higher mobility than their counterparts grown at high temperatures (HT‐MAPbBr_3_). These results encouraged us to explore the VEC method for the growth of all PSC types. Ultimately, we showed that the VEC technique provides a universal strategy for preparing high‐quality PSCs.

## 2 Results and Discussion

### 2.1 Nucleation and Growth Mechanism Using VEC

Supersaturation plays a critical role in driving the nucleation and crystal growth of the MHP precursors.^[^ ^15^, ^25^ ^]^ Consequently, the ability to accurately control precursor supersaturation is an effective strategy for producing high‐quality PSCs. To this effect, we designed a VEC method that does not include the use of temperature gradients and auxiliary substances. The VEC method first entails the preparation of a supersaturated MHP precursor using a low‐pressure solvent evaporation technique under constant temperature conditions. Unlike the traditional processes, which are slower and include steps such as temperature conduction and auxiliary substance diffusion, in the VEC process the pressure can rapidly reach a predetermined value throughout the chamber, enabling more precise control over MHP precursor supersaturation. Rapid and accurate regulation of the system pressure is easily achievable within the confined space and facilitates the fabrication of high‐quality PSCs.

**Figure 1:**
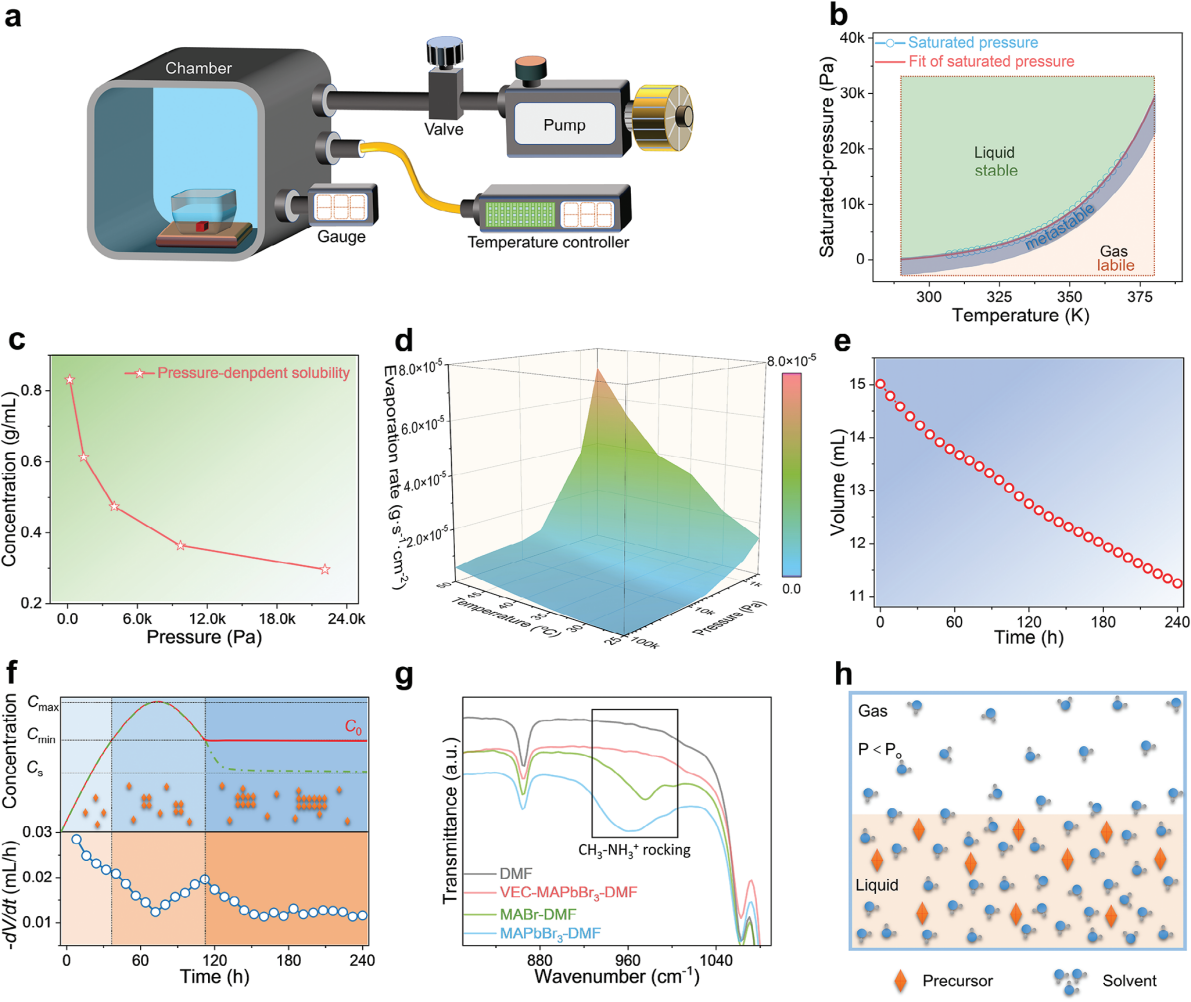
Growth of perovskite single crystals. a) Schematic representation of the VEC apparatus with the crystallization vial placed in an enclosed chamber. b) Phase diagram of DMF, with the liquid and gas phases separated by the *P*–*T* curve. c) Pressure‐dependent solubility of MAPbBr_3_ in DMF. d) Net evaporation rate of the perovskite solution (1 m MAPbBr_3_ in DMF) at different pressures and temperatures. e) Volume of the solution as a function of time. f) La Mer diagram and the solvent evaporation rate −d*V/*d*t* as a function of time. g) FTIR results for different samples: pristine DMF, VEC ‐MAPbBr_3_‐DMF, MABr‐DMF and MAPbBr_3_‐DMF. h) Diagrammatic representation of the evaporation of the solvent when the chamber pressure *P* is lower than the saturated vapor pressure *P*_o_ of the solution.

**Figure** **1**a shows a diagram of the experimental setup that was used to grow PSCs using the VEC method. The vacuum pump and pressure controller were used to lower the pressure in the crystal‐growth chamber to the appropriate level. As the chamber pressure decreases, the solvent within the MHP precursor evaporates to accumulate in the space above the solution. To maintain constant pressure throughout the chamber, the vacuum pump continuously removed excess evaporated solvent vapor from the chamber to trigger the spontaneous nucleation of MHP in the supersaturated solution. Under these conditions, the PSCs grew continuously in the consistently supersaturated environment. Throughout the process of growing the PSCs, the sole task for the experimenter was to control the chamber pressure. Additional details regarding the crystal‐growth chamber are provided in the Experimental section.

The impact of the chamber pressure on the solubility of the MAPbBr_3_ precursor was systematically examined to clarify the effect of the pressure on the crystallization process. When the chamber pressure was lowered to a level below the pressure at which the solvent was saturated, the solvent molecules continually transitioned from the liquid to the gaseous state. Figure 1b shows the phase diagram of the solvent *N*,*N*‐dimethylformamide (DMF), which depicts the two phases separated by the pressure–temperature (*P–T*) curve that was obtained by fitting the saturated pressure data reported for DMF.^[^ ^26^ ^]^ The gas (labile) and liquid (stable) phases reside above and below the *P*–*T* curves, respectively. Throughout nucleation and crystal growth, controlling the pressure and temperature within the chamber in the area adjacent to the *P–T* curve (highlighted in blue and representing the metastable region) effectively regulates the thermodynamic solubility of metastable MHP. Measurement of the temperature‐dependent solubility of MAPbBr_3_ in DMF (Figure S1, Supporting Information) enabled the calculation of its pressure‐dependent solubility, as shown in Figure 1c. The solubility of MAPbBr_3_ in DMF decreased sharply as the saturated pressure increased from 0.1 to 5 kPa, followed by a slow decline as the saturated pressure further increased to 25 kPa. These results indicate that the MAPbBr_3_ PSCs grew faster at low rather than at high saturated pressures. According to the Clausius‐Clapeyron law,^[^ ^27^, ^28^ ^]^ the natural logarithm of the saturated pressure (ln*P*) is a linear function of the reciprocal of the temperature (1/*T*). The linear relationship (details described in Equation S1, Supporting Information) can be expressed as ln *P* =  *B*(1 − 1/*T*), where *B* is a constant characteristic of the substance. Consequently, adjusting the chamber pressure enabled nucleation and crystal growth of the PSCs at lower temperatures.

The solvent evaporation rate significantly influenced the nucleation and crystal growth processes of the PSCs. In our study, the evaporation rate was predominantly influenced by the chamber pressure and temperature. Figure 1d shows a 3D data diagram of the evaporation rate of the MAPbBr_3_ solution at various temperatures and pressures. Details of the method and the corresponding data that were generated by measuring the evaporation rate are presented in the experimental section and Table S1 (Supporting Information), respectively. Additionally, Figures S2 and S3 (Supporting Information) depict the temperature‐ and pressure‐dependent evaporation rates. Notably, the evaporation rate gradually increased as the pressure decreased from 100 to 5 kPa, followed by a marked increase once the pressure decreased below 5 kPa. At constant chamber pressure, the evaporation rate is known to increase linearly as the temperature rises from 25 °C to 50 °C.^[^ ^29^, ^30^ ^]^ These results were well aligned with the above pressure‐ and temperature‐dependent solubility data, as shown in Figure 1c and Figure S1 (Supporting Information). The advantages of the VEC method, which requires neither temperature gradients nor the addition of auxiliary substances, is that the method circumvents the problems caused by thermal‐gradient‐induced convection or solvent conversion, which facilitates the control of nucleation and growth. To achieve single crystals of the highest quality, it is imperative to maximize the relative amount of crystal growth while minimizing ancillary nucleation. The crystal growth rate is known to have an important effect on the quality of the resulting single crystals.^[^ ^15^, ^18^, ^25^ ^]^ An in‐depth analysis of the crystallization kinetics and crystal growth rates is provided in the following sections to more clearly understand the practical observations. The evaporation rate derived from the Hertz‐Knudsen equation can be described as^[^ ^31^ ^]^

(1) $$J\;=\;a\left(P_{s}-P_{r}\right)\sqrt{\frac{M}{2\pi RT}}$$

where *J* is the evaporation rate per unit area of the liquid‐vapor interface, *a* is the fraction of the accessible surface area, *P_s_* is the saturated pressure at a certain temperature, *P*_r_ is the real chamber pressure, *M* is the molecular mass of the solvent, *R* is the ideal gas constant, and *T* is the temperature. By controlling the pressure using the vacuum pump, *P*_r_ was maintained below *P*_s_, which led to continuous evaporation and gradually increased the concentration of the solution. The direct force responsible for driving the interfacial evaporation is the difference between *P*_r_ and *P*_s_, which is the determining step in the crystal growth. The VEC method requires *P*_r_ to be set to a constant value by using the vacuum pump to adjust the pressure. The evaporation rate is therefore mainly determined by *P*_s_. According to Raoult's law^[^ ^32^ ^]^ *P*_s_ of non‐ideal solutions can be described as $P_{s}=\;xyP_{s}^{\prime }$, where *x* is the mole fraction of the solvent, *y* is the activity coefficient of the solvent, and $P_{s}^{\prime }$ is the reference saturated pressure of the solvent in the ideal solution. The evaporation rate varies with the solution concentration because *P*_s_ is determined by *x*. Figure 1e shows the change in the volume of the solution (MAPbBr_3_ in DMF) with evaporation time. Fitting and calculation of the experimental data (Figure 1e) allowed the evaporation rate (d*V/*d*t*) of the solvent to be derived (Figure 1f). Continuous evaporation of the solvent caused the concentration of the solution to gradually increase, subsequently resulting in a decrease in the mole fraction of the solvent. Consequently, this led to a reduction in the *P*_s_ value and a corresponding deceleration of d*V/*d*t*. As shown in Figure 1f, the change in d*V/*d*t* coincides well with the three stages of the La Mer diagram^[^ ^33^ ^]^ similar to those of previous studies.^[^ ^23^ ^]^ In the first stage, the d*V/*d*t* value of solvent decreased as the concentration of the solution increased. In the second stage, the maximum concentration of the solution *C*_max_ was reached, accompanied by the corresponding minimum d*V/*d*t*. In the third stage, as the concentration of the solution decreased and approached a constant value, *C*_0_, d*V/*d*t* initially decreased before gradually stabilizing.

The rate of PSC growth in the MHP solution was determined by the supersaturation of the solution. During the crystal growth stage, the concentration of the solution was kept constant at *C*_0_, that is, the supersaturation of the solution was a stable constant. The volume of the solution decreased as DMF continued to be extracted. Thus, the crystal growth rate (d*m/*d*t*) as a function of the volume of the solution can be expressed as

(2) $$\frac{dm}{dt}=-C\cdot M_{p}\cdot \frac{d}{dt}\left(V_{t1}-V_{t0}\right)$$

where *V* is the solution volume, *C* is the molar concentration of the solution, and *M*_p_ is the molar weight of MAPbBr_3_, respectively. Equation (1) can also be rewritten as

(3) $$J=\frac{\rho }{SM}\cdot \frac{d}{dt}\left(V_{t1}-V_{t0}\right)$$

where *S* is the effective evaporation area, and ρ is the constant density of the solvent. Substituting Equations (1) and (3) into Equation (2) yields

(4) $$\frac{dm}{dt}=-\frac{\mathrm{CSM}M_{p}}{\rho }\cdot J=-\left(P_{s}-P_{r}\right)\cdot \frac{a\mathrm{CSM}M_{p}}{\rho }\cdot \sqrt{\frac{M}{2\pi RT}}$$

According to Equation (4), the crystal growth rate is proportional to the evaporation of the solvent and is eventually controlled by the real chamber pressure *P*_r_. Therefore, the crystal growth rate can be effectively adjusted by controlling the real chamber pressure to obtain high‐quality single crystals. Based on this theoretical analysis, we effectively controlled the number of grains in each batch (inset of Figure S3, Supporting Information).

The effective use of the VEC method for producing PSCs requires perovskite to be completely absent from the volatile components. This was verified by using Fourier transform infrared spectroscopy (FTIR) to analyze the volatile components (Figure 1g): DMF (pristine DMF), VEC‐MAPbBr_3_‐DMF (volatiles condensed during the VEC process), MABr‐DMF (MABr dissolved in DMF), and MAPbBr_3_‐DMF (MAPbBr_3_ dissolved in DMF). The full FTIR spectrum is shown in Figure S4 (Supporting Information). Notably, the peak positions of VEC‐MAPbBr_3_‐DMF were completely aligned with those of pristine DMF.^[^ ^26^ ^]^ However, in the case of MABr‐DMF and MAPbBr_3_‐DMF, the distinct peak in the region 900–1000 cm^−1^ was ascribed to CH_3_‐NH_3_^+^ (MA^+^) rocking vibrations.^[^ ^34^, ^35^ ^]^ This result demonstrates that MA^+^ did not evaporate with DMF at room temperature, indicating that the composition of the volatiles remained unchanged at low pressure. This implies that the solvent used during the VEC process is easily recoverable for reuse. The results also showed that the solvent in the homogeneous solution evaporated first because of its lower saturation pressure and weaker thermal stability compared to those of the solute, as shown in Figure 1h. Consequently, we selected suitable evaporation rate parameters (temperature and pressure) for the perovskite precursors with specific components to enable nucleation and growth to be more precisely controlled.

**Figure 2:**
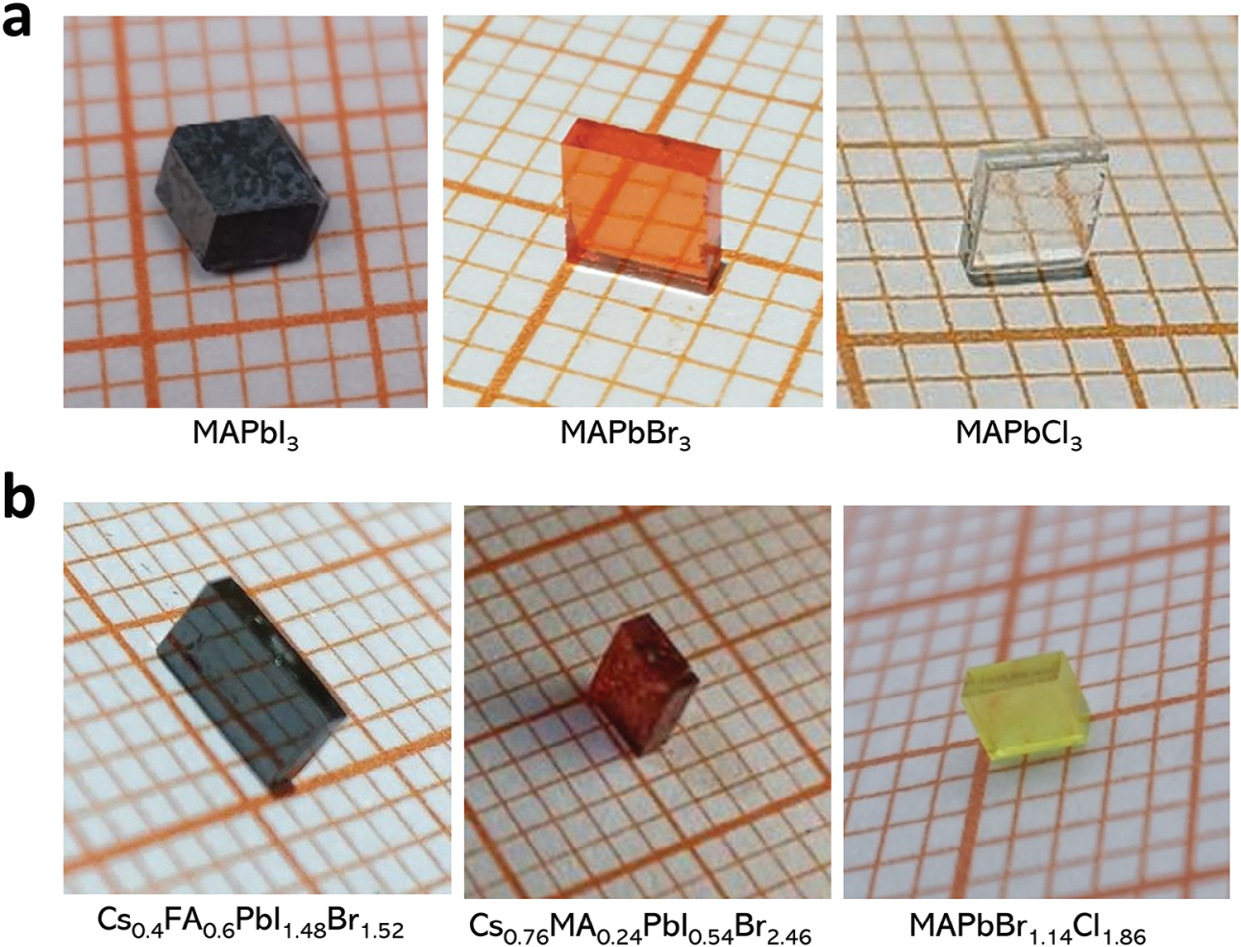
Photographic images of MHP single crystals. a) MAPbX_3_ (X = I, Br, Cl) single crystals. b) Cs_0.4_FA_0.6_PbI_1.48_Br_1.52_, Cs_0.76_MA_0.24_PbI_0.54_Br_2.46_ and MAPbI_1.14_Br_1.86_ single crystals.

**Figure** **2**a shows the three PSCs—MAPbI_3_, MAPbBr_3_, and MAPbCl_3_—that were grown using the VEC method. Most PSCs, including mixed organic cations (Cs*_x_* FA_1‐_ *_x_* PbI*_y_* Br_1‐_ *_y_*, Cs*_x_* MA_1‐_ *_x_* PbI*_y_* Br_1‐_ *_y_*), mixed halide anions (MAPbI*_x_* Br_3‐_ *_x_*), fully inorganic compounds (CsPbBr_3_), and low‐dimensional (BA_2_PbI_2_Br_2_) single crystals, can be grown using the VEC method, as depicted in Figure 2b and Figure S5 (Supporting Information). The sizes of the PSCs are listed in Table S2 (Supporting Information). Their X‐ray diffraction (XRD) patterns, as well as powder diffraction patterns are shown in Figures S6 and S7 (Supporting Information), respectively. The elemental compositions of the PSCs were measured using energy dispersive spectroscopy (EDS),^[^ ^36^ ^]^ as shown in Figure S8 (Supporting Information). The experimental conditions, including the pressure and temperature, are presented in Table S3 (Supporting Information). The successful growth of a series of PSCs demonstrated the capability, universality, and flexibility of the VEC method.

### 2.2 Characterization of High‐Quality MAPbBr_3_ Single Crystals

**Figure 3:**
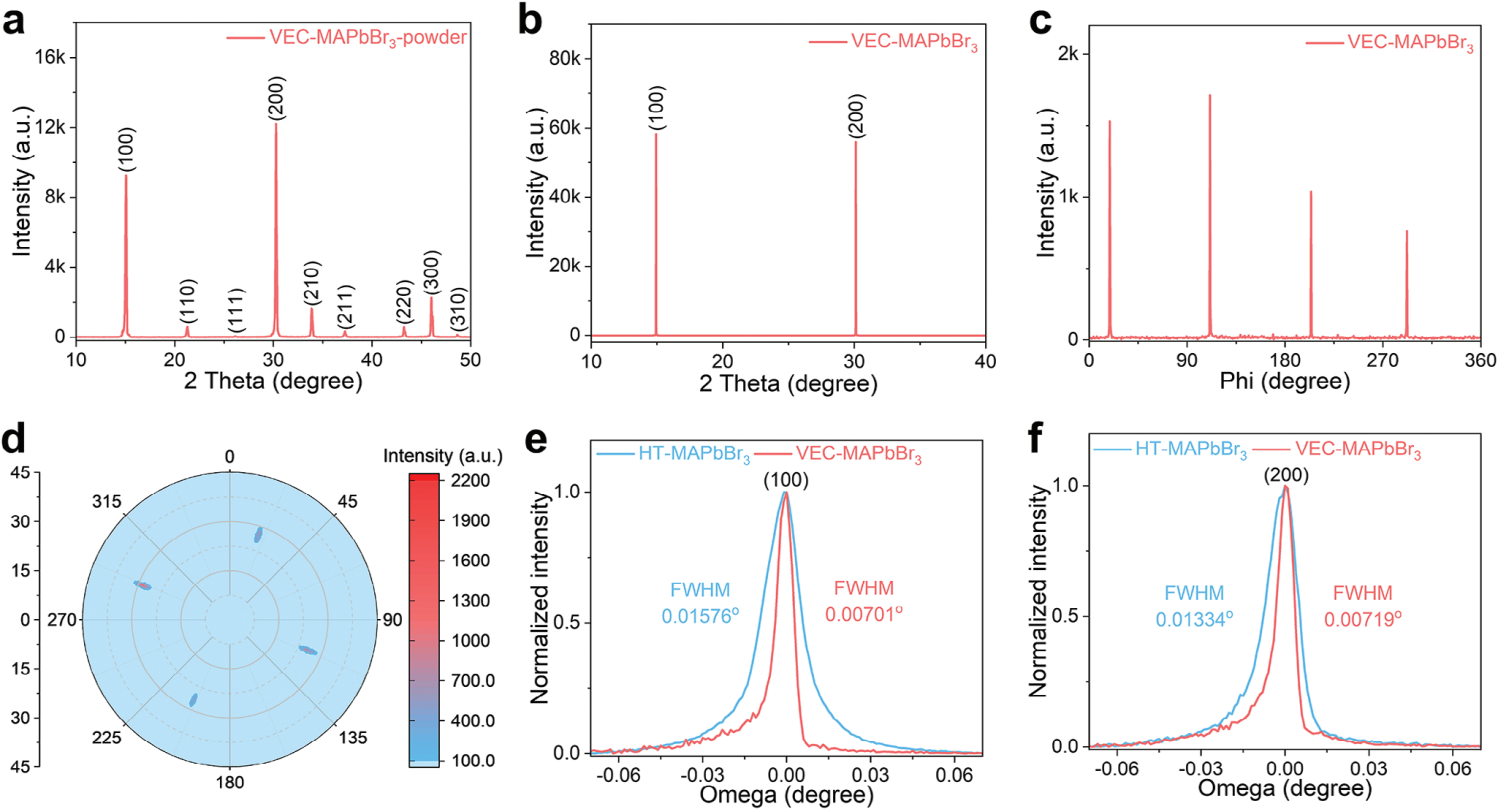
X‐ray diffraction patterns of MAPbBr_3_ single crystals. a) Power XRD of ground powder of VEC‐MAPbBr_3_ single crystal (VEC‐MAPbBr_3_‐powder). b) XRD 2*θ* scan of the maximal facet of the VEC‐MAPbBr_3_ single crystal. c) Phi‐scan pattern and d) pole figure of the (201) diffraction peak of the VEC‐MAPbBr_3_ single crystal. High‐resolution XRD rocking curve of the e) (100) and f) (200) diffraction peaks of the VEC‐MAPbBr_3_ and HT‐MAPbBr_3_ (grown by the HT method) single crystals.

Changes in the nucleation and crystal growth kinetics of PSCs affect their crystallinity. The crystal structure of the MAPbBr_3_ single crystals grown using the VEC method was probed using X‐ray diffraction (XRD). For comparison, MAPbBr_3_ single crystals that were grown using the high‐temperature (HT) method were also investigated.^[^ ^13^ ^]^ First, XRD analysis of a powdered single crystal confirmed the crystal structure of MAPbBr_3_ to be cubic. As shown in **Figure** **3**a, the powder XRD pattern of the MAPbBr_3_ single crystal grown via the VEC method has diffraction peaks at 15.06°, 21.28°, 30.26°, 33.88°, 37.26°, 43.24°, and 46.14°, which correspond to the (100), (110), (200), (210), (211), (220), and (300) planes, in good agreement with the cubic phase of MAPbBr_3_.^[^ ^18^, ^37^ ^]^ In addition, the X‐ray 2*θ* scan of the maximal facet of the single crystal revealed only those diffraction peaks that correspond to the corresponding planes (Figure 3b), suggesting that the MAPbBr_3_ single crystal was well structured. To characterize the in‐plane domain properties, the (201) diffraction peak of the VEC‐MAPbBr_3_ single crystal was subjected to a phi‐scan between 0° and 360°. The phi‐scan yielded four well resolved peaks 90° apart, as shown in Figure 3c, and these results demonstrated the fourfold symmetry of the crystal. The fact that only well resolved diffraction lines are observed indicated the high crystalline quality of the in‐plane structure. The orientation and order of the crystals were further investigated and confirmed by pole figure measurements, as shown in Figure 3d. In this investigation, the sample was rotated around the phi‐axis from 0° to 360° in steps of 1° and tilted step‐by‐step around the chi‐axis from 0° to 45°, again in steps of 1° at fixed 2θ values. The appearance of four spots 90° apart in the pole figure demonstrates that the crystal is both in‐plane and out‐of‐plane, which validates the results of the phi‐scan analysis. In addition, the (100) and (200) peaks were further examined carefully using high‐resolution X‐ray rocking curve analysis by assigning a 2*θ* value of 15.06° to the (100) and 30.26° to the (200) planes, as shown in Figure 3e,f. The full width at half maximum (FWHM) values of these two peaks are as narrow as 0.00701° and 0.00719° for the (001) and (002) peaks, respectively. These values are much smaller than those of the crystals grown using the HT method, which have FWHMs as wide as 0.01576° and 0.01334°, respectively. To the best of our knowledge, this FWHM of 0.00701° is the narrowest among the values of all reported MAPbBr_3_ single crystals.^[^ ^15^, ^17^, ^37^, ^38^ ^]^ Furthermore, the FWHM analysis of each reflection provided the lattice strain of both crystals using the tangent formula. The larger FWHM values of HT‐MAPbBr_3_ crystals indicate that these crystals experience increased lattice strain. This leads to a higher defect concentration and nonradiative recombination processes to ultimately affect the overall crystalline quality of the single crystals.^[^ ^39^, ^40^ ^]^ Table S4 (Supporting Information) summarizes the FWHM and lattice strain values of the VEC‐MAPbBr_3_ and HT‐MAPbBr_3_ single crystals. These results agree with the trend in the literature, and demonstrate the superior single‐crystal quality of our crystals and the excellent performance of the VEC method for growing perovskite single crystals.

**Figure 4:**
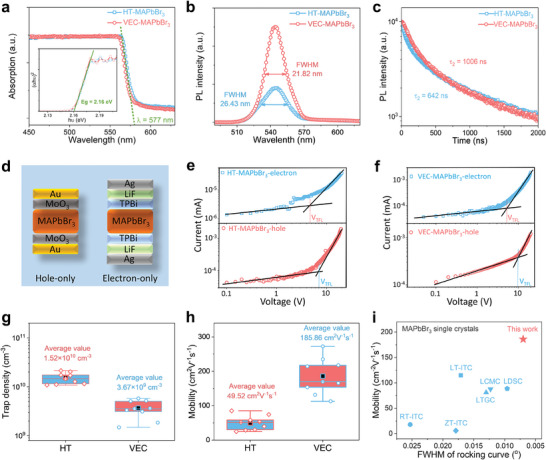
Optical and optoelectronic properties of MAPbBr_3_ single crystals. a) Absorption spectra of VEC‐MAPbBr_3_ and HT‐MAPbBr_3_ single crystals. Inset: corresponding Tauc plots displaying the extrapolated optical band gaps. b) Photoluminescence spectra of VEC‐MAPbBr_3_ and HT‐MAPbBr_3_ single crystals at the same excitation intensity. c) Time‐resolved photoluminescence of VEC‐MAPbBr_3_ and HT‐MAPbBr_3_ single crystals. d) Device architecture of the hole‐only and electron‐only MAPbBr_3_ single crystal devices. Dark current‐voltage curves of hole‐only and electron‐only devices for the e) VEC‐MAPbBr_3_ and f) HT‐MAPbBr_3_ single crystals. Statistics of g) calculated trap density and h) hole carrier mobility for both the VEC‐MAPbBr_3_ and HT‐MAPbBr_3_ single crystals. i) Comparison of rocking curve and mobility of the MAPbBr_3_ single crystals. Details of each data point and the corresponding citation are summarized in Table S6 (Supporting Information).

Typically, crystals with improved crystallinities exhibit lower defect densities. In our next experiment, we investigated the optical properties of the MAPbBr_3_ single crystals. The absorption spectra of VEC‐MAPbBr_3_ and HT‐MAPbBr_3_ single crystals are shown in **Figure** **4**a. Both single crystals exhibited a sharp absorption onset at 577 nm, suggesting a low density of in‐gap defect states. The optical bandgap of 2.16 eV, estimated from the Tauc plots, corresponds with the previously reported value for the same single crystal grown using other methods.^[^ ^13^, ^18^ ^]^ The PL peaks of the VEC‐MAPbBr_3_ and HT‐MAPbBr_3_ single crystals appear at 547 nm, as shown in Figure 4b. The PL peak of VEC‐MAPbBr_3_ has a narrower FWHM of 21.82 nm compared to that of HT‐MAPbBr_3_. The PL intensity of the VEC‐MAPbBr_3_ single crystal was higher than that of the HT‐MAPbBr_3_ single crystal, indicating that the former crystal was of a higher quality than the latter crystal. The recombination dynamics of the photoexcited species in both the VEC‐MAPbBr_3_ and HT‐MAPbBr_3_ single crystals were investigated using time‐resolved photoluminescence (TRPL) spectroscopy, as shown in Figure 4c. The photoluminescence decay of the VEC‐MAPbBr_3_ single crystal was slower (τ_2_ ≈ 1006 ns) than that of the HT‐MAPbBr_3_ single crystal (τ_2_ ≈ 642 ns) and these values are related to the internal recombination mechanism of the crystals.^[^ ^16^, ^25^ ^]^ Furthermore, the carrier lifetimes varied in response to changes in the measurement position, excitation power, and crystalline plane,^[^ ^41^, ^42^ ^]^ as illustrated in Figure S9 (Supporting Information). In summary, the carrier lifetime of the VEC‐MAPbBr_3_ single crystal was significantly longer compared with that of the HT‐MAPbBr_3_ single crystal, indicating a much lower recombination rate and fewer defect densities. These results again demonstrate that the VEC‐MAPbBr_3_ single crystal is of high quality.

In general, an improvement in the quality of the perovskite crystal leads to improved electrical properties. To investigate the electronic properties of the MAPbBr_3_ PSCs, we fabricated hole‐only and electron devices (Figure 4d) to calculate the trap density (*n*_trap_) and mobility (μ_h_). The space‐charge‐limited current (SCLC) is the most common method for calculating these parameters for PSCs.^[^ ^13^, ^18^ ^]^ We employed a pulsed voltage sweep SCLC method to minimize the ion migration effect during the measurement to accurately determine the μ_h_ and *n*_trap_ values of MAPbBr_3_ PSCs.^[^ ^43^, ^44^ ^]^ We utilized this method to obtain the dark current‐voltage (*I*‐*V*) characteristics of the MAPbBr_3_ devices from 0 to 20 V in increments of 50 mV. A short‐duration voltage pulse (10 ms), followed by a long rest time at 0 V (10 s) was used for this measurement. Figure 4e,f shows a kink in the *I–V* curve for both the VEC‐MAPbBr_3_ and HT‐MAPbBr_3_ devices. Typical *I*–*V* characteristics can be divided into three regimes: Ohmic, trap‐filled limit (TFL), and Child regions. In our work, at low bias voltages, the *I*–*V* curve followed Ohm's law, which was confirmed by the linearly dependent fit owing to the conductivity of the background charge carriers. As the bias voltage increased, a trap‐filled region started at *V*_TFL_, where the current increased rapidly to the TFL, indicating that all available trap states were filled by carriers. *n*_trap_ was calculated using the following equation^[^ ^45^ ^]^

(5) $$n_{\mathrm{traps}}=\frac{2\varepsilon \varepsilon_{0}V_{\mathrm{TFL}}}{eL^{2}}\;$$

where *ε* is the relative dielectric constant of perovskite (= 25.5), ε_0_ is the vacuum permittivity, *e* is the electronic charge, and *L* is the thickness of the single crystal. Using Equation (5), we evaluated the trap density of the crystals grown using both the HT and VEC techniques. The determined average trap density is 1.52  ×  10^10^ cm^−3^ for HT‐MAPbBr_3_, whereas it is only 3.67  ×  10^9^ cm^−3^ for VEC‐MAPbBr_3_, as summarized in Figure 4g and Table S5 (Supporting Information, the statistics from single crystals with different thicknesses). The trap density of the VEC‐MAPbBr_3_ single crystal is significantly lower than that of its microcrystalline thin‐film (10^17^ cm^−3^) and that of commercial semiconductors, such as CdTe (10^11^−10^13^ cm^−3^), CIGS (10^13^ cm^−3^), and Si (10^13^−10^14^ cm^−3^).^[^ ^15^, ^46^, ^47^, ^48^ ^]^

In addition, we fitted the *I*–*V* curve to calculate the mobility. Once all available trap states are filled, the current is assumed to follow the Mott‐Gurney law in the absence of trapping. The hole carrier mobility μ_h_ is calculated using the Mott‐Gurney law:^[^ ^21^ ^]^

(6) $$J_{D}=\frac{9\varepsilon \varepsilon_{0}\mu_{h}V^{2}}{8L^{3}}\;$$

where *J*_D_ represents the dark current density, and *V* denotes the bias voltage. Using Equation (6), we conservatively estimated the hole carrier mobility. Surprisingly, the average hole carrier mobility of the VEC‐MAPbBr_3_ single crystal is as high as 185.86 cm^2^ V^−1^ s^−1^, more than 3 times higher than that of the HT‐MAPbBr_3_ single crystal (49.52 cm^2^ V^−1^ s^−1^). These results are summarized in Figure 4h and Table S5 (Supporting Information, the statistics from single crystals with different thickness). The essential properties of MAPbBr_3_ single crystals that were grown using various methods at different temperatures are summarized in Figure 4i and Table S6 (Supporting Information).^[^ ^13^, ^15^, ^16^, ^17^, ^38^, ^49^, ^50^ ^]^ The ultranarrow rocking curve and ultrahigh mobility of the VEC‐MAPbBr_3_ single crystals attest to their excellent crystal quality, which is critical for realizing high‐performance optoelectronic applications.

## 3 Conclusion

In summary, we reported a universal and effective VEC method suitable for growing high‐quality MHP single crystals. This method relies on the regulation of solvent volatilization by adjusting the chamber pressure, which allows the crystallization and growth of MHP single crystals to be precisely grown in solution. Using the VEC method, we successfully grew a range of perovskite single crystals, including MAPbX_3_ (X = I, Br, Cl), mixed organic cations (Cs*_x_* FA_1‐_ *_x_* PbI*_y_* Br_1‐_ *_y_*, Cs*_x_* MA_1‐_ *_x_* PbI*_y_* Br_1‐_ *_y_*), mixed halide anions (MAPbI*_x_* Br_3‐_ *_x_*), fully inorganic (CsPbBr_3_), and low‐dimensional (BA_2_PbI_2_Br_2_) perovskite single crystals. The new method, which circumvents the problem of temperature fluctuation and the need to introduce additives, facilitates the growth of high‐quality MAPbBr_3_ single crystals characterized by exceptional crystallinity (rocking curve FWHM ≈ 0.00701°), a low trap density (3.67 × 10^9^ cm^−3^), and an ultrahigh carrier mobility of 185.86 cm^2^ V^−1^ s^−1^.

## 4 Experimental Section

### Chemicals and Reagents

The solvents γ‐butyrolactone (GBL), *N*, *N*‐dimethylformamide (DMF), and dimethyl sulfoxide (DMSO) were purchased from Sigma Aldrich. Lead iodide (PbI_2_), lead bromide (PbBr_2_), lead chloride (PbCl_2_), cesium bromide (CsBr), formamidinium iodide (FAI), methylammonium iodide (MAI), methylammonium bromide (MABr), methylammonium chloride (MACl), and n‐butylammonium iodide (BAI) were purchased from the Xi'an Polymer Light Technology Corp. All other chemicals were purchased from Aladdin or Sinopharm Chemical Reagent Co., Ltd. All the materials were used as received without further purification.

### Experimental Setup for Crystal Growth via VEC

As shown in Figure 1a and Figure S10a (Supporting Information), the crystal‐growth equipment consisted mainly of a customized stainless‐steel cubic vacuum chamber (64 × 64 × 64 cm). Several flange interfaces were designed and mounted on the walls of the chamber, to which the temperature and pressure controllers were attached for data collection, environmental parameter control, maintenance, and monitoring. For precise and dependable pressure control and measurement, an electronic mass flow meter (Figure S10b, Supporting Information), an oil‐free vacuum pump (XDS35i, Edwards Ltd., Flintshire, UK) (Figure S10c, Supporting Information), a pressure controller (946 Vacuum System Controller, MKS Instruments, Andover, MA, USA) (Figure S10d, Supporting Information), and a pressure sensor (model: KJL300808, MKS Instruments, Andover, MA, USA) (Figure S10e, Supporting Information) were utilized to dynamically regulate the chamber pressure (≥ 0.1 Pa) within the crystal growth chamber. Temperature control and measurements were performed using a customized heating and cooling stage (Figure S10f, Supporting Information) installed inside the chamber. This stage was connected to an mK1000 temperature controller (Figure S10g, Supporting Information) and a liquid‐nitrogen pump (Figure S10h, Supporting Information) via an electronic feedthrough and two gas feedthroughs (Figure S10i, Supporting Information).

### VEC Growth of the Perovskite Single Crystals

For the VEC method, AX (FAI, BAI, and CsX) and PbX_2_ (X = I, Br, and Cl) were dissolved at appropriate molar ratios in organic solvents at different concentrations for single‐crystal growth. The solution was maintained at room temperature and stirred overnight. Finally, the precursor solution was filtered using a polytetrafluoroethylene filter with a pore size of 0.22 µm. The prepared precursor solution was then placed in a crystal growth chamber (the equipment was custom‐designed and ‐built in the laboratory) at an appropriate pressure and room temperature for crystallization.

### Growth of MAPbBr_3_ Single Crystals at High Temperature

In the HT method, MABr and PbBr_2_ were dissolved at a 1:1 molar ratio in DMF as the solvent. To ensure that the components were completely dissolved, the solution was stirred overnight at a specific temperature. The precursor solution was filtered using a polytetrafluoroethylene filter with a 0.22 µm pore size. Then, the prefabricated solution was placed on a hot plate and the temperature was increased to 80 °C.

### Solubility Test

MAPbBr_3_ powder was prepared by grinding a large piece of a single crystal into a fine powder in an agate mortar. DMF (5 mL) was placed in a vial on a hot plate and stirred. While keeping the temperature of the DMF constant, a small amount of MAPbBr_3_ powder was added in 0.005 g increments until the solution reached saturation. Saturation conditions were assumed to have been reached when the MAPbBr_3_ no longer completely dissolved within 30 min of addition to the DMF. This process was repeated at various temperatures.

### Evaporation Rate Test

A high‐precision electronic mass scale was placed in the crystal growth chamber and a quartz Petri dish with an inner diameter of 5 cm was placed on the scale. To maintain the required temperature and pressure in the chamber, 10 mL of sample (DMF or solution) was added to the quartz Petri dish. Electronic scale mass data were read hourly. The sample volatilization rate was equal to the ratio of the mass change to the product of the evaporation area and time.

### Characterization

The FTIR spectra were recorded on a Spectrum II infrared spectrometer in the 400–4000 cm^−1^ wavenumber range at a resolution of 2 cm^−1^. Powder X‐ray diffraction (XRD) was performed using an X‐ray diffractometer (D8‐advance, Bruker) with Cu‐Kα radiation in the 2*θ* range of 5°−80°. The high‐resolution X‐ray diffraction measurement was performed using an X'Pert MRD with Cu‐K_α1_ radiation at *V* = 40 kV and *I* = 20 mA. The VEC‐MAPbBr_3_ single crystal was characterized by XRD 2θ scans, phi‐scans, omega scans, and pole figure measurements. The UV–Vis absorbance spectrum was acquired at room temperature using a PerkinElmer Lambda 950 UV–Vis–NIR spectrophotometer in reflection mode with an integrating sphere attachment operating in the 200−700 nm region. Steady‐state and time‐resolved photoluminescence measurements of the MAPbBr_3_ single crystals were performed using an FLS‐920 fluorescence spectrometer. Dark *I–V* curves were recorded using a Keithley 2400 instrument. The device was maintained in the dark at room temperature. A nonlinear response was observed and analyzed according to SCLC theory. The device area is 0.04 cm^2^.

## Conflict of Interest

The authors declare no conflict of interest.

## Supporting information

Supporting Information

## Data Availability

The data that support the findings of this study are available in the Supporting Information of this article.
