# Supplementary material for: Perovskite Single Crystals by Vacuum Evaporation Crystallization
Source: Adv Sci (Weinh). 2024 Mar 29;11(22):2400150. doi: 10.1002/advs.202400150 (PMC11165535; doi:10.1002/advs.202400150)
Supplement: Supplementary file 1 — Supporting Information [file ADVS-11-2400150-s001.pdf]

ADVANCED SCIENCE
Open Access

Supporting Information

for *Adv. Sci.*, DOI 10.1002/advs.202400150

Perovskite Single Crystals by Vacuum Evaporation Crystallization

Dong Liu, Xianyuan Jiang, Hao Wang, Hao Chen, Ying-Bo Lu, Siyu Dong, Zhijun Ning, Yong Wang, Zhongchen Wu* and Zongcheng Ling*

Supporting Information

Perovskite Single Crystals by Vacuum Evaporation Crystallization

Dong Liu, Xianyuan Jiang, Hao Wang, Hao Chen, Ying-Bo Lu, Siyu Dong, Zhijun Ning, Yong Wang, Zhongchen Wu*, and Zongcheng Ling*

D. Liu, Prof. Y. B. Lu, Prof. Y. Wang, Prof. Z. Wu, Prof. Z. Ling
School of Space Science and Physics, Institute of Space Sciences, Shandong University, Weihai 264209, China.
Email: z.c.wu@sdu.edu.cn

X. Jiang, Prof. Z. Ning
School of Physical Science and Technology, ShanghaiTech University, Shanghai 201210, China.
Email: ningzhj@shanghaitech.edu.cn

H. Wang, S. Dong
One State Key Laboratory of High Field Laser Physics and CAS Center for Excellence in Ultra-Intense Laser Science, Shanghai Institute of Optics and Fine Mechanics, Chinese Academy of Sciences, Shanghai 201800, China. Center of Materials Science and Optoelectronics Engineering, University of the Chinese Academy of Sciences, Beijing 100049, China.

H. Chen
The Edward S. Rogers Department of Electrical and Computer Engineering, University of Toronto, Toronto, Ontario M5S 3G4, Canada.

Keywords: perovskite single crystals, crystallization, crystal growth, low pressure, vacuum evaporation

Table of Contents

Supplementary equations

Equation S1…………………………………………………………………….3

Supplementary Figures

Figure S1………………………………………………………………………5

Figure S2………………………………………………………………………6

Figure S3………………………………………………………………………7

Figure S4………………………………………………………………………8

Figure S5………………………………………………………………………9

Figure S6……………………………………………………………………… …10

Figure S7…………………………………………………………... ……….11

Figure S8………………………………………………………..……..……. 12

Figure S9………………………………………………………..……..……. 13

Figure S10……………………………………………………..……..……14

Supplementary Tables

Table S1………………………………………………………………………15

Table S2………………………………………………………………………16

Table S3………………………………………………………………………17

Table S4………………………………………………………………………18

Table S5………………………………………………………………………19

Table S6………………………………………………………………………20

References

References……………………………………………………………….21

Equation S1

To determine the temperature dependence of the saturated pressure (P) of a fluid along the liquid-vapor coexistence curve, most chemistry and physics textbooks employ the Clausius-Clapeyron equation:[1-4]

$$\ln\frac{P}{P_0} = A\left(\frac{1}{T_0} - \frac{1}{T}\right) \qquad (1)$$

where P_0 and T_0 are the vapor pressure and absolute temperature of a reference point on the coexistence curve, respectively, and A is a constant characteristic of the substance. Equation (1) is typically derived from the integration of the Clapeyron equation for vaporization[5]

$$\frac{dP}{dT} = \frac{\Delta H}{T(V_g - V_L)} \qquad (2)$$

under the following assumptions: (i) the volume occupied by one mole of liquid under its saturated-pressure is negligible compared to the volume occupied by one mole of vapor, $V_L \ll V_g$, (ii) the vapor behaves as an ideal gas, $V_g = RT/P$, and (iii) ΔH of vaporization does not change with temperature, $\Delta H \approx$ constant. These are reasonably good approximations at low temperatures where the vapor pressure is small.

The Clausius-Clapeyron equation, Equation (1), can predict the vapor pressure of some fluids along the entire coexistence curve with good accuracy, even near the critical point, where the approximations made for the integration of Equation (2) are not valid. To simplify this understanding, we use the compressibility factor $Z = PV/RT$ and rewrite Equation (2) as follows [6]

$$\frac{d\ln P}{d(1/T)} = -\frac{\Delta H}{R\Delta Z} \qquad (3)$$

where ΔZ is the difference between the compressibilities of the vapor and liquid phases. Far enough from the critical point the above-mentioned approximations hold, so that $\Delta Z \approx 1$ and $\Delta H \approx$ constant, and integration of Equation (3) leads to equation (1). Furthermore, although ΔZ and ΔH are dependent on the temperature, owing to a compensating effect, the ratio $\Delta H/\Delta Z$ in Equation (3) is constant for some substances over the entire temperature range from the triple point to the critical point. Then, by assuming that the right-hand side of Equation (3) is constant, the integration of this equation using the critical point as a reference yields

$$\ln P = B\left(1 - \frac{1}{T}\right) \qquad (4)$$

where B is a constant characteristic of the substance. Although Equations (1) and (4) provide the natural logarithm of the vapor pressure as a linear function of $1/T$, we note that they have been obtained using

different approximations.

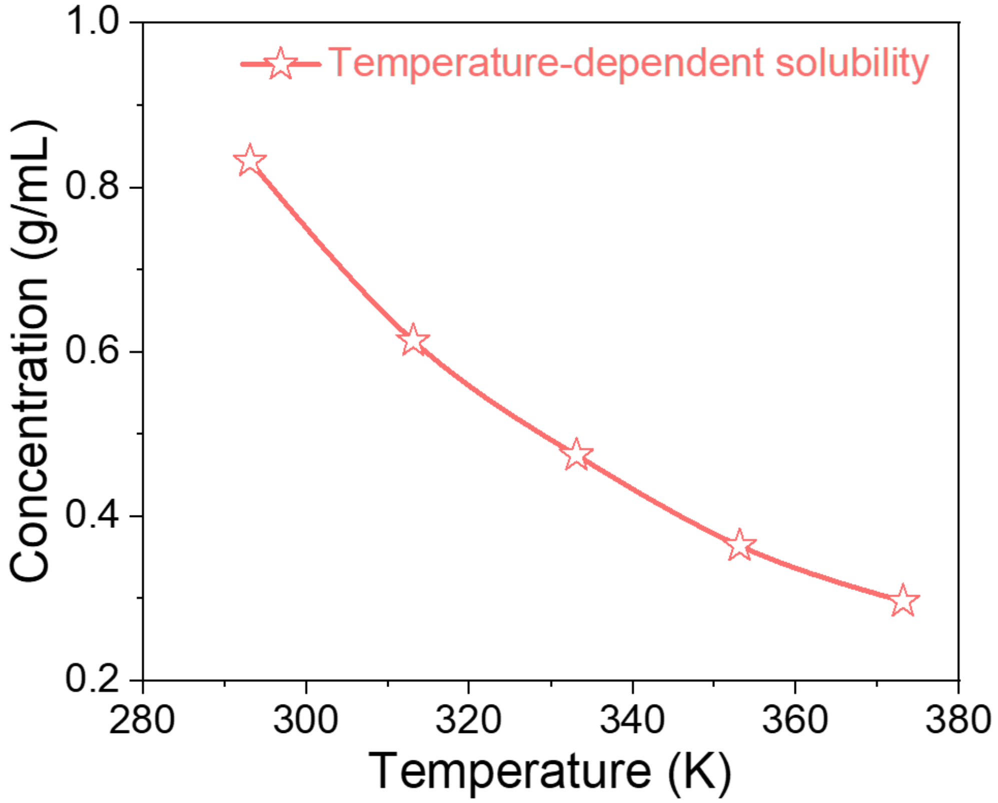

Figure S1. Temperature-dependent solubility of $MAPbBr_3$ in DMF.

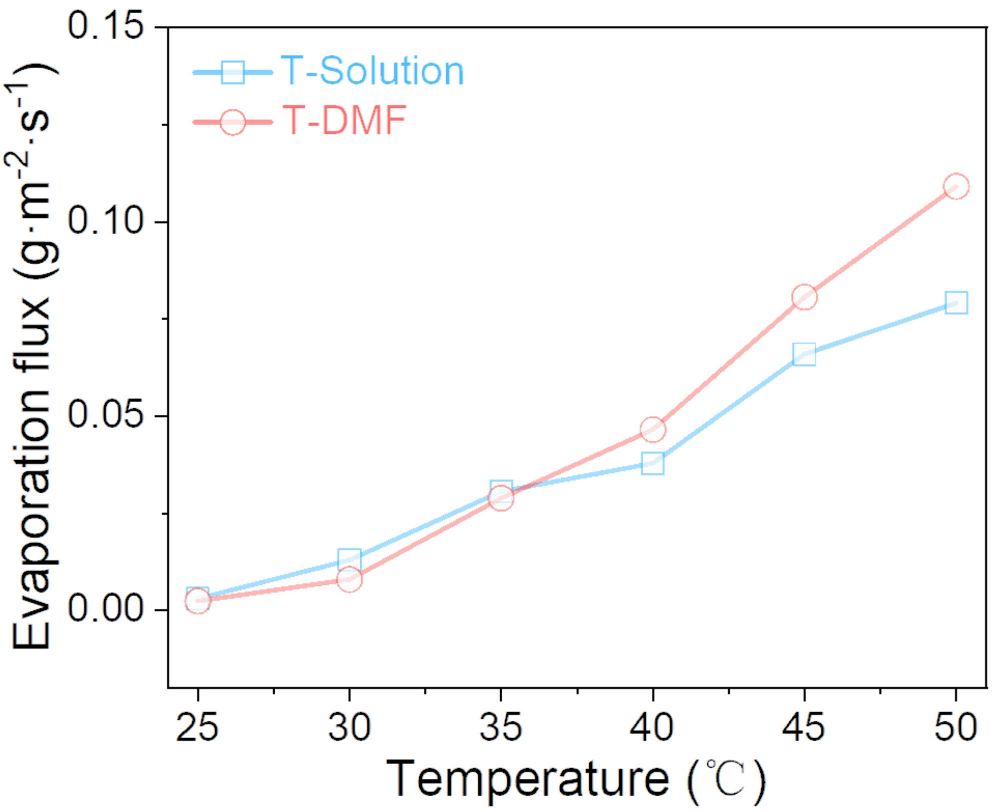

Figure S2. Temperature-dependent evaporation rate of DMF and solution (1 M $MAPbBr_3$ in DMF).

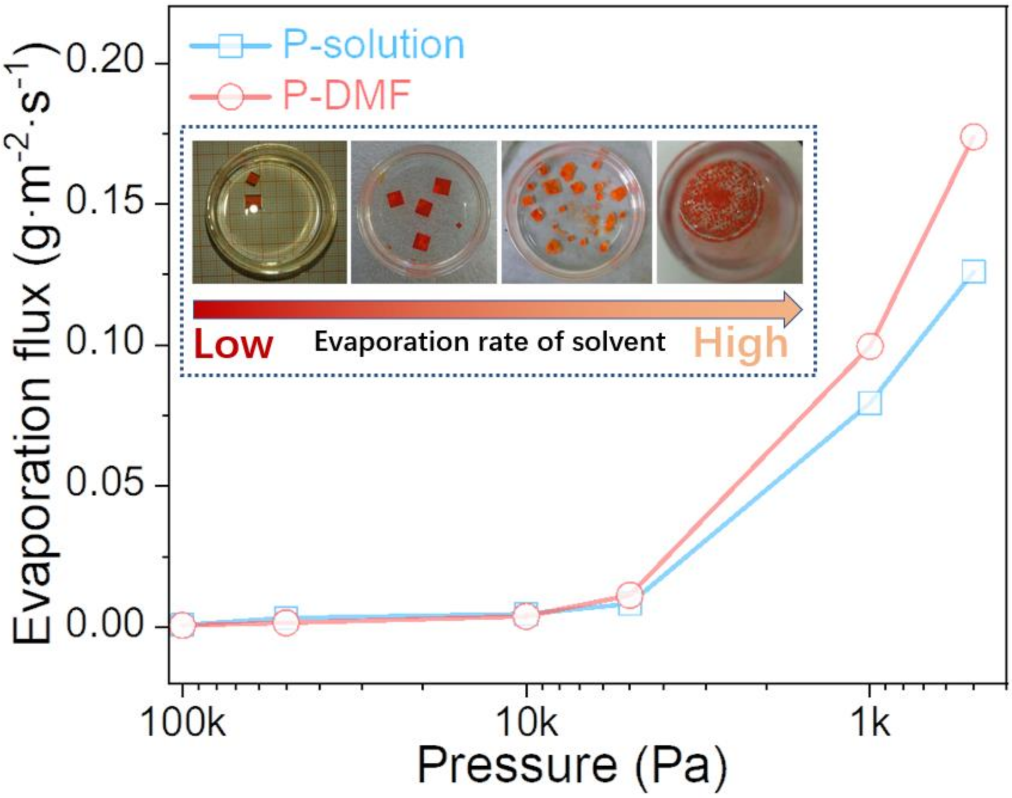

Figure S3. Pressure-dependent evaporation rate of DMF and solution (1 M $MAPbBr_3$ in DMF). Inset: Photographs of $MAPbBr_3$ single crystal growth with varying solvent evaporation rates.

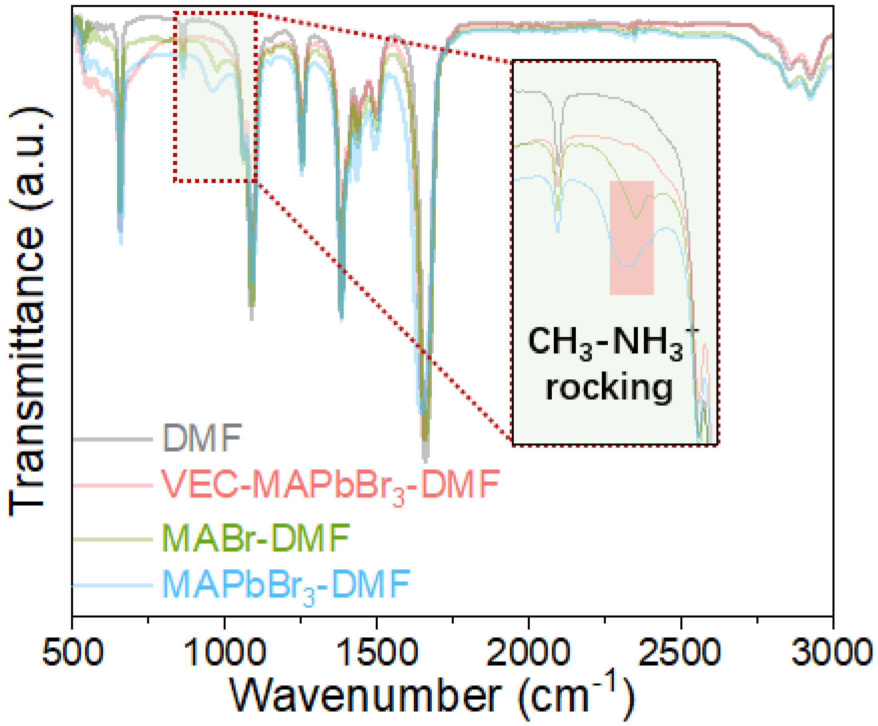

Figure S4. Full FTIR spectra of different samples: pristine DMF, VEC-$MAPbBr_3$-DMF, MABr-DMF and $MAPbBr_3$-DMF.

.

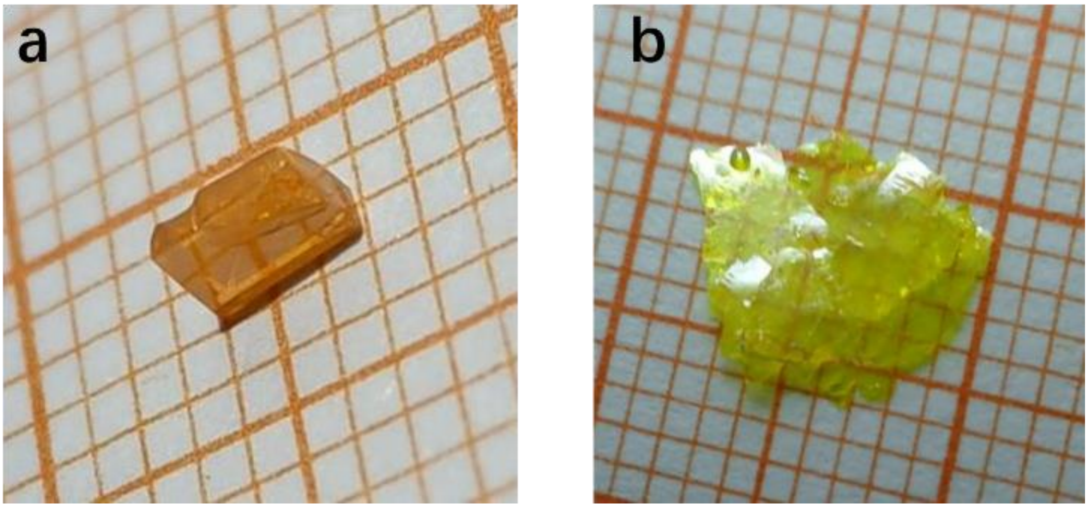

Figure S5. Photographs of perovskite single crystals: (a) fully inorganic ($CsPbBr_3$) and (b) two-dimensional (2D) ($BA_2PbI_2Br_2$) single crystals.

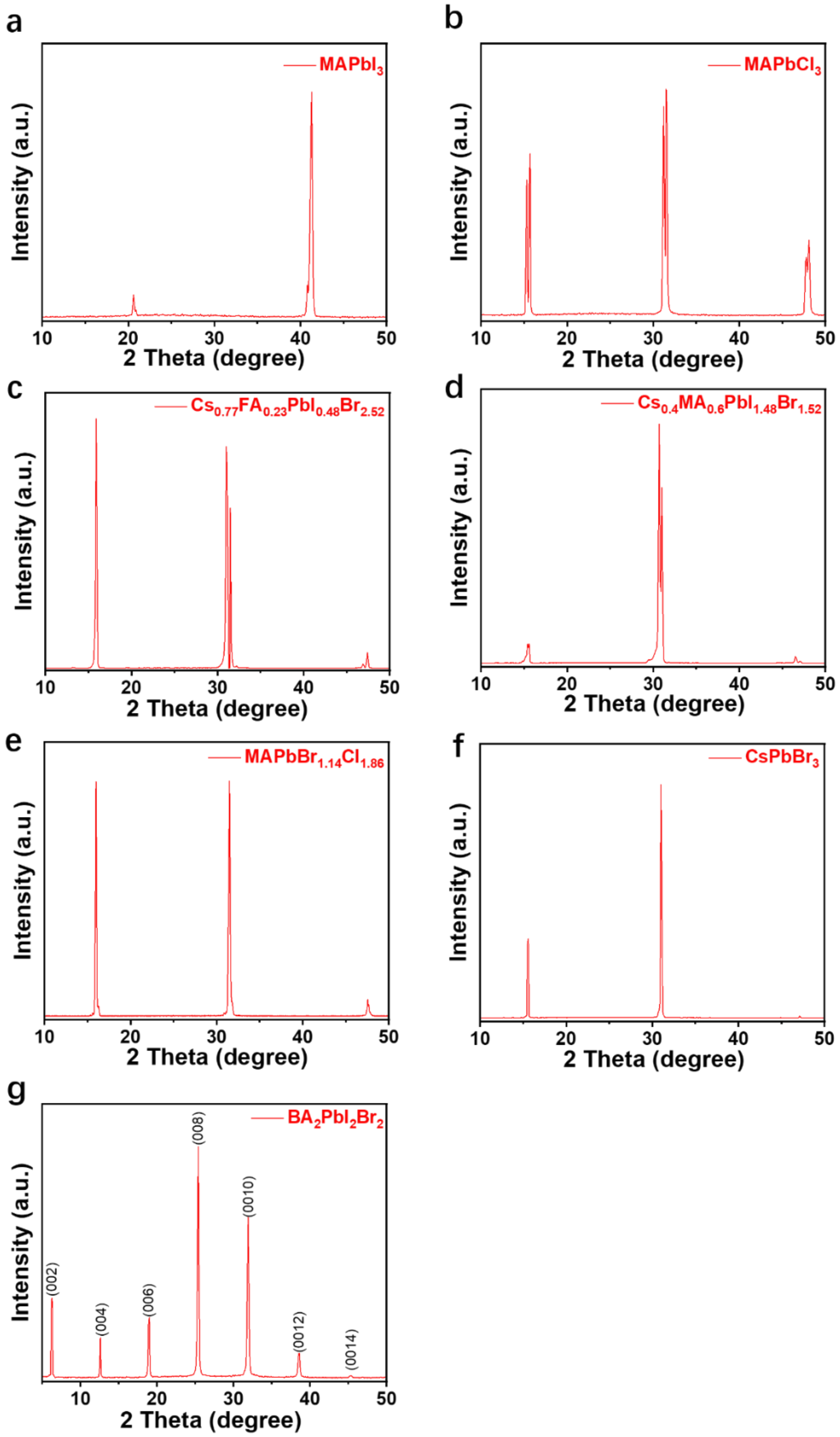

Figure S6. XRD 2θ scans of a single crystal of $MAPbI_3$ (a), $MAPbCl_3$ (b), $Cs_{0.77}FA_{0.23}PbI_{0.48}Br_{2.52}$ (c), $Cs_{0.4}MA_{0.6}PbI_{1.48}Br_{1.52}$ (d), $MAPbBr_{1.14}Cl_{1.86}$ (e), $CsPbBr_3$ (f), and $BA_2PbI_2Br_2$ (g).

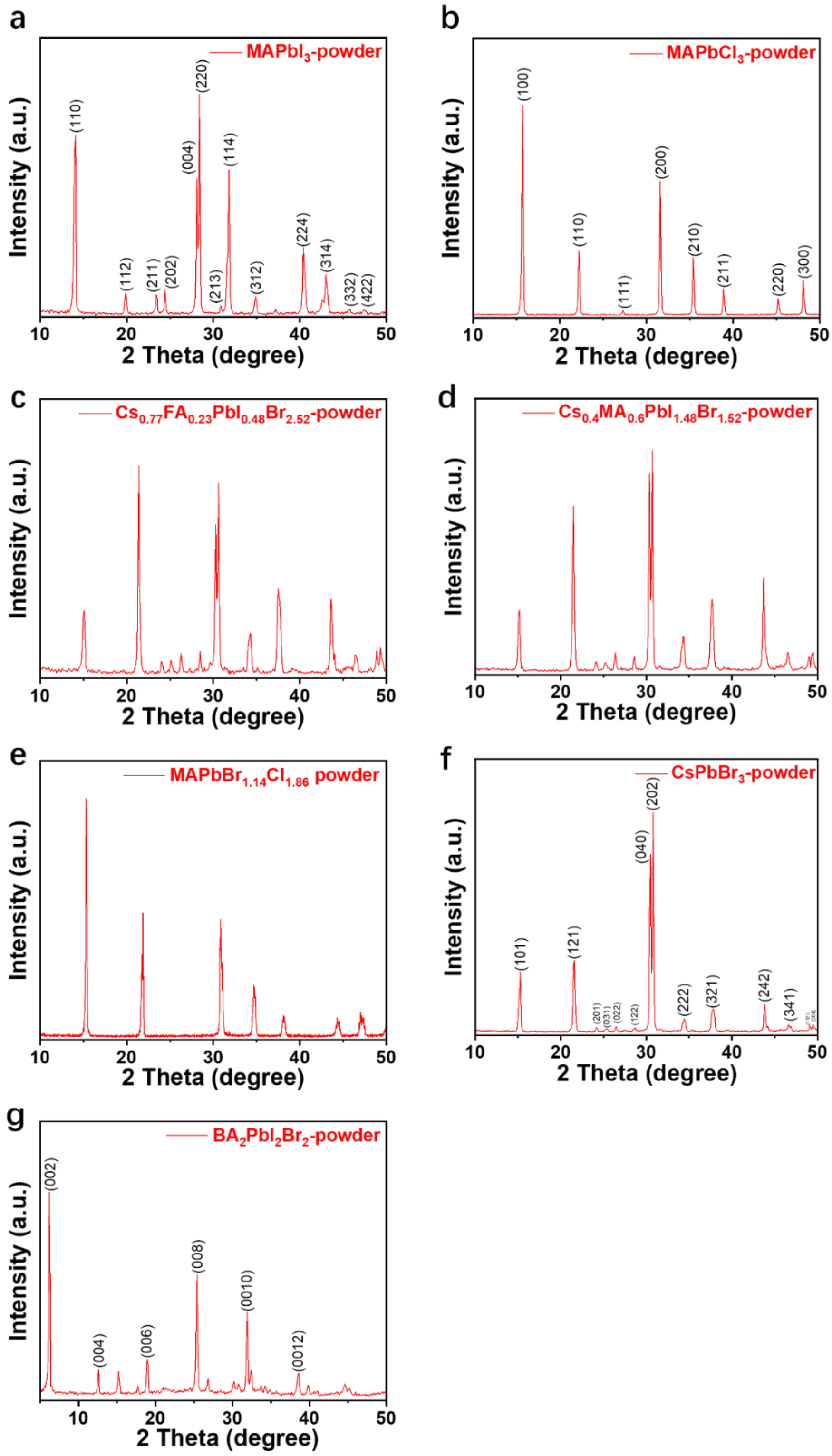

Figure S7. XRD 2θ scans of a powder sample of each of $MAPbI_3$ (a), $MAPbCl_3$ (b), $Cs_{0.77}FA_{0.23}PbI_{0.48}Br_{2.52}$ (c), $Cs_{0.4}MA_{0.6}PbI_{1.48}Br_{1.52}$ (d), $MAPbBr_{1.14}Cl_{1.86}$ (e), $CsPbBr_3$ (f), and $BA_2PbI_2Br_2$ (g).

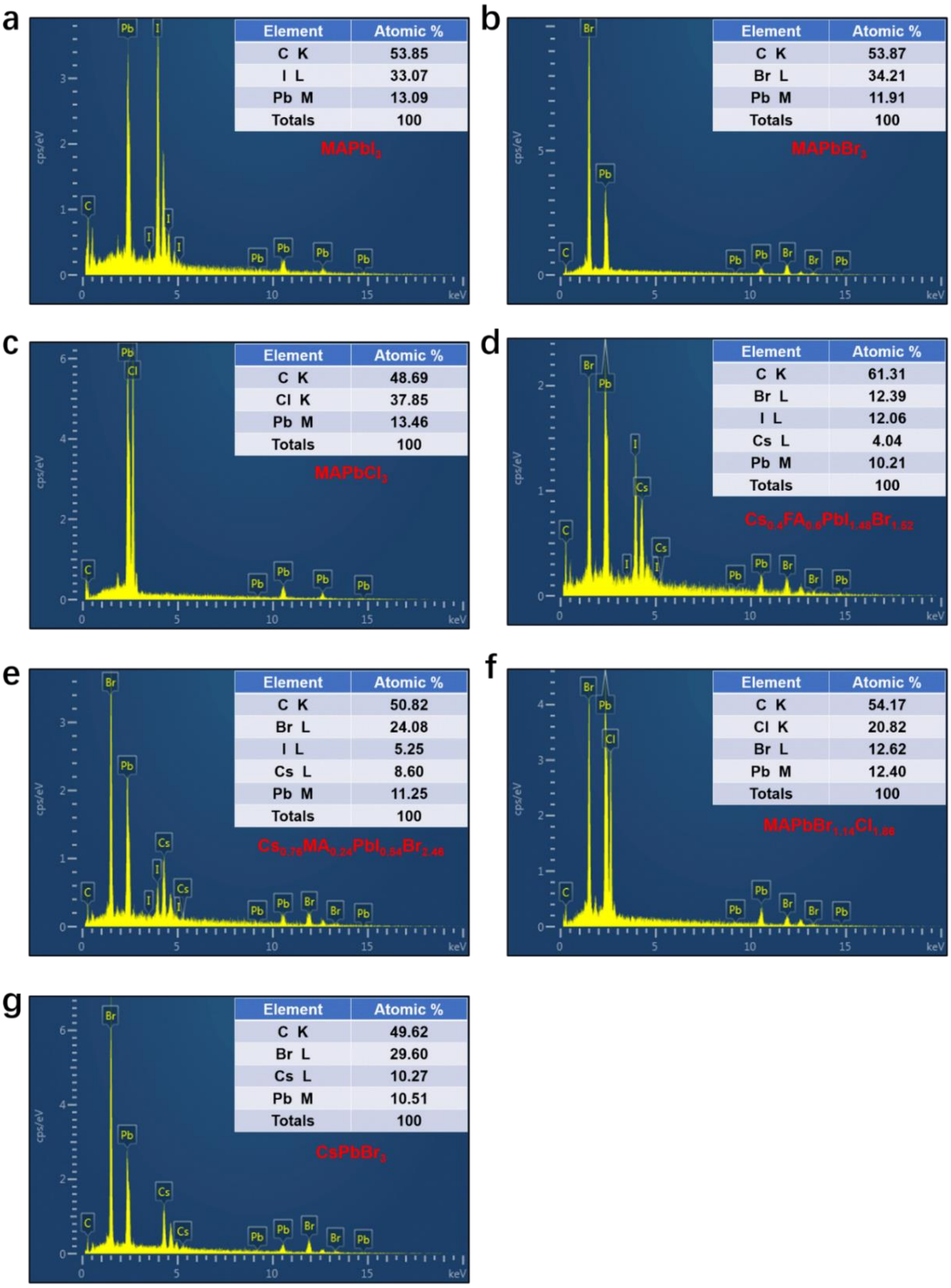

Figure S8. Elemental compositions determined by EDS analyses of a single crystal of each of: (a) $MAPbI_3$, (b) $MAPbBr_3$, (c) $MAPbCl_3$, (d) $Cs_{0.4}FA_{0.6}PbI_{1.48}Br_{1.52}$, (e) $Cs_{0.76}MA_{0.24}PbI_{0.54}Br_{2.46}$, (f) $MAPbBr_{1.14}Cl_{1.86}$, and (g) $CsPbBr_3$.

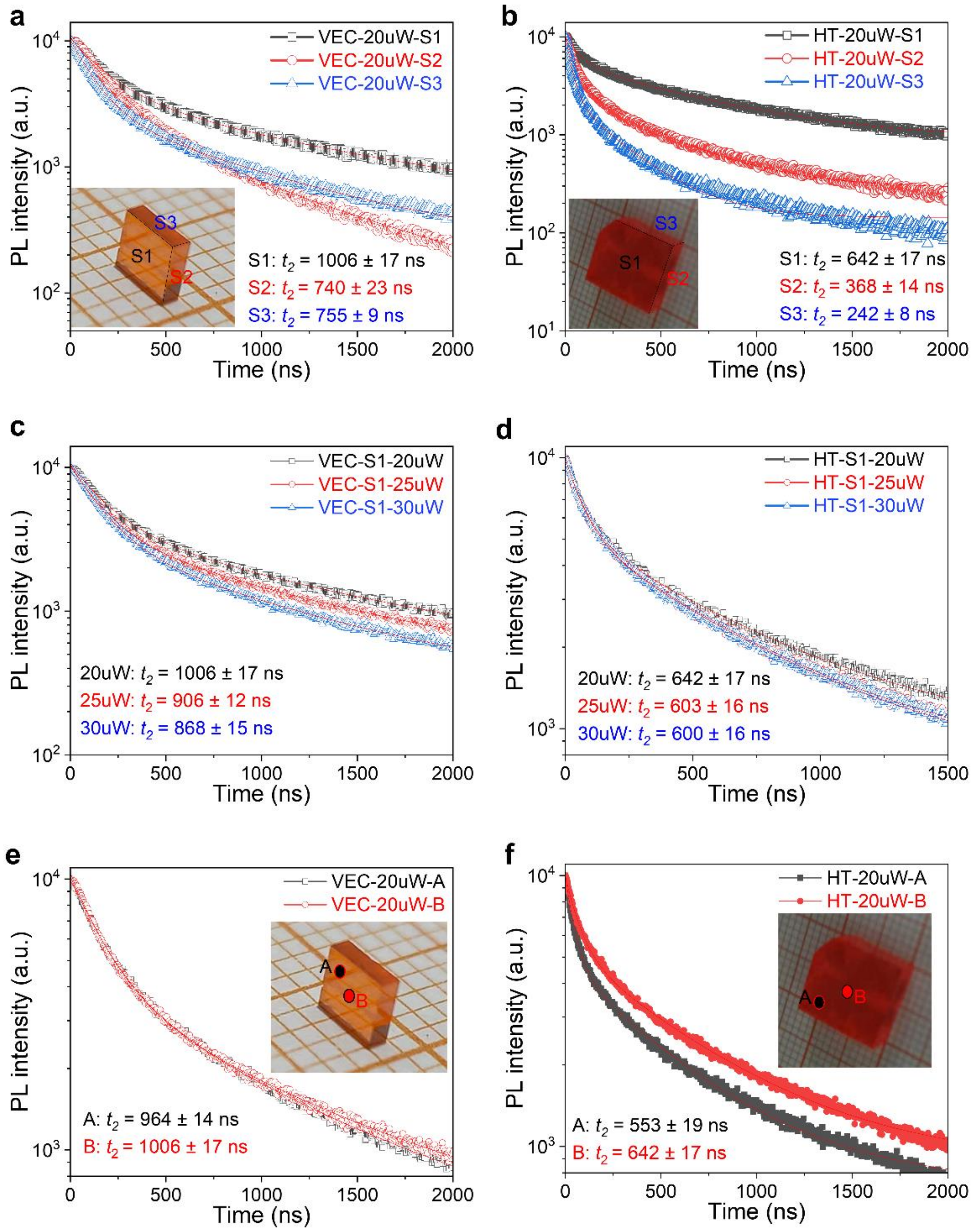

Figure S9. PL decay times of (a) VEC-$MAPbBr_3$ and (b) HT-$MAPbBr_3$ single crystals on different crystalline planes (S1, S2, and S3), (c) VEC-$MAPbBr_3$ and (d) HT-$MAPbBr_3$ single crystals using different excitations (20 μW, 25 μW, and 30 μW), and of (e) VEC-$MAPbBr_3$ and (f) HT-$MAPbBr_3$ single crystals at different positions (at points A and B).

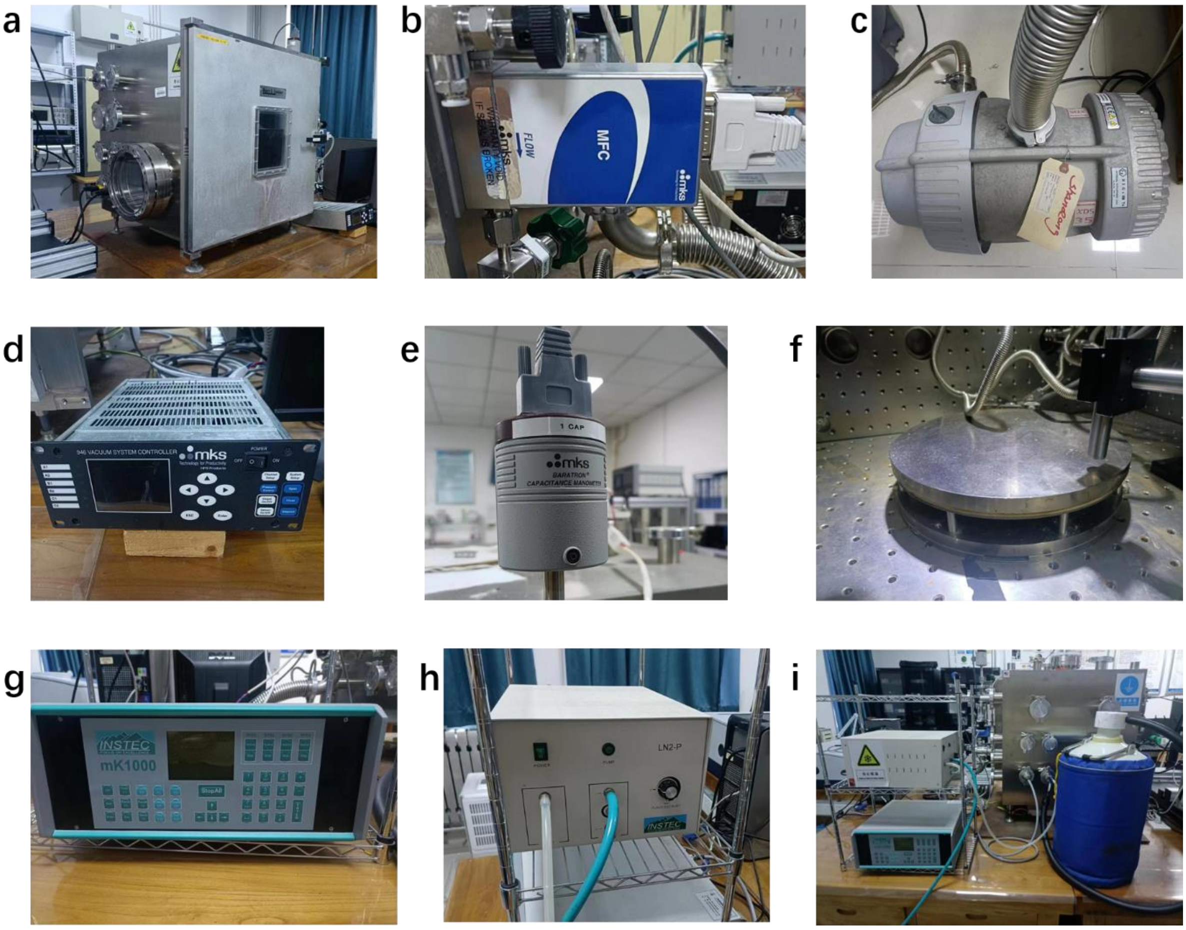

Figure S10. Photographs of (a) the customized stainless box-shaped vacuum chamber, (b) the electronic mass flow meter, (c) the oil-free vacuum pump (XDS35i, Edwards Ltd., Flintshire, UK), (d) the gas pressure controller (946 Vacuum System Controller, MKS Instruments, Andover, MA, USA), (e) the pressure sensor (model: KJL300808, MKS Instruments, Andover, MA, USA), (f) customized heating and cooling stage, (g) mK1000 temperature controller, (h) liquid nitrogen pump, and (i) one electronic feedthrough and two gas feedthroughs.

Table S1. Detailed experimental data of the evaporation rate (ER, g s^{-1} cm^{-2}) of $MAPbBr_3$ solution at various temperatures (T, °C) and pressures (P, kPa). The evaporation rate for each combination of temperature and pressure was determined by averaging three measurements.

P / ER(g s^{-1} cm^{-2}) / T	0.5 kPa	1 kPa	5 kPa	10 kPa	50 kPa	100 kPa
25 °C	$1.424\pm0.027\times10^{-5}$	$7.964\pm0.103\times10^{-6}$	$1.251\pm0.013\times10^{-6}$	$5.392\pm0.068\times10^{-7}$	$1.081\pm0.023\times10^{-7}$	$3.969\pm0.023\times10^{-8}$
30 °C	$2.154\pm0.045\times10^{-5}$	$1.321\pm0.021\times10^{-5}$	$2.028\pm0.026\times10^{-6}$	$1.211\pm0.013\times10^{-6}$	$6.590\pm0.090\times10^{-7}$	$2.998\pm0.022\times10^{-7}$
35 °C	$3.430\pm0.027\times10^{-5}$	$1.843\pm0.021\times10^{-5}$	$3.672\pm0.050\times10^{-6}$	$2.670\pm0.005\times10^{-6}$	$1.77\pm0.026\times10^{-6}$	$1.044\pm0.008\times10^{-6}$
40 °C	$3.945\pm0.064\times10^{-5}$	$2.192\pm0.027\times10^{-5}$	$4.652\pm0.053\times10^{-6}$	$3.758\pm0.058\times10^{-6}$	$2.945\pm0.034\times10^{-6}$	$2.291\pm0.018\times10^{-6}$
45 °C	$5.362\pm0.057\times10^{-5}$	$2.535\pm0.033\times10^{-5}$	$6.715\pm0.058\times10^{-6}$	$5.468\pm0.057\times10^{-6}$	$4.136\pm0.052\times10^{-6}$	$3.695\pm0.024\times10^{-6}$
50 °C	$7.202\pm0.074\times10^{-5}$	$3.781\pm0.041\times10^{-5}$	$9.884\pm0.121\times10^{-6}$	$7.860\pm0.072\times10^{-6}$	$6.673\pm0.065\times10^{-6}$	$5.494\pm0.034\times10^{-6}$

Table S2. Sizes of the MHP single crystals in Figure 2c and Figure S4.

	MHP single crystal	Size: L×W×H (mm×mm×mm)
Figure 2a	$MAPbI_3$	4×4×2
	$MAPbBr_3$	3×1×3
	$MAPbCl_3$	2.5×0.6×2.5
Figure 2b	$Cs_{0.4}FA_{0.6}PbI_{1.48}Br_{1.52}$	4×1×2
	$Cs_{0.76}MA_{0.24}PbI_{0.54}Br_{2.46}$	2×1×2
	$MAPbBr_{1.14}Cl_{1.86}$	2.5×1×2.5
Figure S4	$CsPbBr_3$	3×1×2
	$BA_2PbI_2Br_2$	9×8×N

Table S3. Additional details of the growth processes for each type of MHP single crystal.

MHP single crystals	Solvent	Concentration (g/mL)	Pressure (Pa)	Temperature (°C)
$MAPbI_3$	GBL	0.62	3000	60
$MAPbBr_3$	DMF	0.48	1000	25
$MAPbCl_3$	DMSO	0.34	500	25
$Cs_{0.4}FA_{0.6}PbI_{1.48}Br_{1.52}$	GBL/DMF (v:v = 1:1)	0.24	2000	50
$Cs_{0.76}MA_{0.24}PbI_{0.54}Br_{2.46}$	GBL/DMF (v:v = 1:4)	0.24	1200	50
$MAPbBr_{1.14}Cl_{1.86}$	DMF/DMSO (v:v = 1:1)	0.42	800	25
$CsPbBr_3$	DMSO	0.29	1000	60
$BA_2PbI_2Br_2$	DMF	0.22	2000	50

Table S4. FWHM values and calculated lattice strain corresponding to different XRD reflections for both the VEC-$MAPbBr_3$ and HT-$MAPbBr_3$ single crystals.

Peak position 2θ (degree)	FWHM (degree)		Lattice strain (%)	
	VEC	HT	VEC	HT
15.06	0.00701	0.01576	0.01504	0.02898
30.26	0.00719	0.01334	0.01619	0.02387

Table S5. Trap density and carrier mobility of $MAPbBr_3$ single crystals with different crystal thickness.

Method	Crystal thickness	Trap density (hole-only)	Hole carrier mobility
	mm	cm^{-3}	$cm^2 V^{-1} s^{-1}$
VEC	1.78	5.78×10^9	169.62
VEC	1.78	3.11×10^9	272.40
VEC	1.78	3.56×10^9	234.87
VEC	1.58	5.08×10^9	112.73
VEC	1.58	5.19×10^9	131.61
VEC	1.58	3.61×10^9	214.48
VEC	2.76	3.33×10^9	153.67
VEC	2.76	1.85×10^9	217.65
VEC	2.76	1.48×10^9	165.74
HT	0.5	2.03×10^{10}	58.30
HT	0.5	1.47×10^{10}	51.59
HT	0.5	2.14×10^{10}	30.18
HT	0.9	1.70×10^{10}	28.67
HT	0.9	1.11×10^{10}	39.83
HT	0.9	1.74×10^{10}	24.80
HT	1.28	1.27×10^{10}	52.86
HT	1.28	1.08×10^{10}	85.14
HT	1.28	1.15×10^{10}	74.35

Table S6. Performance metrics of $MAPbBr_3$ single crystals with different growth methods.

Method	Temperature [°C]	Trap density [cm^{-3}]	Lifetime [ns]	FWHM of rocking curve [°]	Mobility [$cm^2\ V^{-1}\ s^{-1}$]	Ref.
AVC	RT	5.8×10^9	$978^{a)}$	-	115	[7]
ITC	80	3.0×10^{10}	$300^{a)}$	-	24	[8]
LTGC	60	6.7×10^9	$815^{b)}$	0.019	83.9	[9]
LDSC	RT	4.4×10^9	$997^{b)}$	0.0096	-	[10]
LDSC	RT	4.5×10^9	$1099^{b)}$	0.0096	88.6	[11]
LCMC	45	2.1×10^9	1126	0.0123	87.8	[12]
RT-ITC		6.59×10^9		0.0253	87.8	[13]
ZT-ITC		3.06×10^9		0.0179	6	[13]
LT-ITC		2.55×10^9		0.0171	115	[13]
VEC	RT	3.67×10^9	$1006^{b)}$	0.00701	185.86	This work

$^{a)}$ Measurement by transient absorption spectroscopy; $^{b)}$ measurement by time-resolved PL spectroscopy.

References

[1] C. H. P. Lupis, *Chemical Thermodynamics of Materials*, Elsevier, New York, **1983**, P 35.

[2] C. E. Reid, *Chemical Thermodynamics*, McGraw-Hill, New York, **1990**, P 73.

[3] M. Bailyn, *A Survey of Thermodynamics*, A. I. P. Press, New York, **1994**; P 265.

[4] R. S. Berry, S. A. Rice, J. Ross, *Physical Chemistry, 2nd ed.* Oxford University Press, Oxford, **2000**, P 659.

[5] O. L. I. Brown, *J. Chem. Educ.* **1951**, *28*, 428.

[6] S. Velasco, F. L. Román, J. A. White, *J. Chem. Educ.* **2009**, *86*, 106.

[7] D. Shi, V. Adinolfi, R. Comin, M. Yuan, E. Alarousu, A. Buin, Y. Chen, S. Hoogland, A. Rothenberger, K. Katsiev, Y. Losovyj, X. Zhang, P. A. Dowben, O. F. Mohanned, E. H. Sargent, O. M. Bakr, *Science* **2015**, *347*, 519.

[8] M. I. Saidaminov, A. L. Abdelhady, B. Murali, E. Alarousu, V. M. Burlakov, W. Peng, I. Dursun, L. Wang, Y. He, G. Maculan, A. Goriely, T. Wu, O. F. Mohammed, O. M. Bakr, *Nat. Commun.* **2015**, *6*, 7586.

[9] Y. Liu, Y. Zhang, Z. Yang, J. Feng, Z. Xu, Q. Li, M. Hu, H. Ye, X. Zhang, M. Liu, K. Zhao, S. F. Liu, *Mater. Today* **2019**, *22*, 67.

[10] F. Yao, J. Peng, R. Li, W. Li, P. Gui, B. Li, C. Liu, C. Tao, Q. Lin, G. Fang, *Nat. Commun.* **2020**, *11*, 1194.

[11] Z. Zhang, H. Li, H. Di, D. Liu, W. Jiang, J. Ren, Z. Fan, F. Liao, L. Lei, G. Li, Y. Xiong, Y. Zhao, *ACS Appl. Electron. Mater.* **2023**, *5*, 388.

[12] Z. Zhu, W. Li, W. Deng, W. He, C. Yan, X. Peng, X. Zeng, Y. Gao, X. Fu, N. Lin, B. Gao, W. Yang. *J. Mater. Chem. C* **2022**, *10*, 6837.

[13] Y. Cho, H. R. Jung, Y. S. Kim, Y. Kim, J. Park, S. Yoon, Y. Lee, M. Cheon, S. Jeong, W. Jo. *Nanoscale* **2021**, *13*, 8275.
